# Time-Intensity Curve Analysis of Contrast-Enhanced Ultrasound for Non-Ossified Thyroid Cartilage Invasion in Laryngeal Squamous Cell Carcinoma

**DOI:** 10.3390/tomography11050057

**Published:** 2025-05-16

**Authors:** Milda Pucėtaitė, Dalia Mitraitė, Rytis Tarasevičius, Davide Farina, Silvija Ryškienė, Saulius Lukoševičius, Evaldas Padervinskis, Valdas Šarauskas, Saulius Vaitkus

**Affiliations:** 1 Department of Radiology, Faculty of Medicine, Medical Academy, Lithuanian University of Health Sciences, A. Mickevičiaus Str. 9, 44307 Kaunas, Lithuania; dalia.mitraite@lsmu.lt (D.M.); silvija.ryskiene@lsmu.lt (S.R.); saulius.lukosevicius@lsmu.lt (S.L.); 2 Department of Radiology, Lithuanian University of Health Sciences Kaunas Clinics, Eivenių 2, 50009 Kaunas, Lithuania; rytis.tarasevicius@kaunoklinikos.lt; 3 Department of Radiological Sciences, University of Brescia, Piazzale Spedali Civili 1, 25123 Brescia, Italy; davide.farina@unibs.it; 4 Department of Otorhinolaryngology, Faculty of Medicine, Medical Academy, Lithuanian University of Health Sciences, A. Mickevičiaus Str. 9, 44307 Kaunas, Lithuania; evaldas.padervinskis@lsmu.lt (E.P.); saulius.vaitkus@lsmu.lt (S.V.); 5 Department of Pathological Anatomy, Faculty of Medicine, Medical Academy, Lithuanian University of Health Sciences, A. Mickevičiaus Str. 9, 44307 Kaunas, Lithuania; valdas.sarauskas@lsmu.lt

**Keywords:** contrast-enhanced ultrasound, laryngeal squamous cell carcinoma, non-ossified thyroid cartilage invasion, diagnostic performance

## Abstract

**Objective:** This study aimed to assess the diagnostic value of contrast-enhanced ultrasound (CEUS) time–intensity curve (TIC) parameters in detecting non-ossified thyroid cartilage invasion in patients with laryngeal squamous cell carcinoma (SCC). **Methods**: A CEUS TIC analysis was performed on 32 cases from 27 patients with histologically confirmed laryngeal SCC. The diagnostic performance of time to peak (TTP), peak intensity (PI), wash-in slope (WIS), area under the curve (AUC), and their quantitative differences (∆TTP, ∆PI, ∆WIS, and ∆AUC) to discriminate between the invaded and the non-invaded non-ossified thyroid cartilage was determined using ROC analysis. A logistic regression analysis was employed to identify significant predictors. **Results**: In an ROC analysis, of all TIC parameters analyzed separately, ∆TTP showed the greatest diagnostic performance (AUC: 0.85). A ∆TTP cut-off of ≤ 8.9 s differentiated between the invaded and the non-invaded non-ossified thyroid cartilage with a sensitivity of 100%, specificity of 76.9%, and accuracy of 81.3%. A combination of ∆TTP and PI increased the AUC to 0.93, specificity to 100%, and accuracy to 96.8%, but reduced the sensitivity to 83.3%. Meanwhile, the visual assessment of enhancement on CEUS to detect cartilage invasion had 83.3% sensitivity and 84.6% specificity. In a univariate logistic regression, only ∆TTP was a significant predictor of non-ossified thyroid cartilage invasion (OR: 0.80; 95% CI: 0.64–1.00). For every second increase in ∆TTP, the probability of thyroid cartilage invasion decreased by 20%. **Conclusions**: CEUS TIC parameters, particularly a combination of ∆TTP and PI, showed high diagnostic performance in the detection of non-ossified thyroid cartilage invasion in laryngeal SCC.

## 1. Introduction

Squamous cell carcinoma (SCC) is the most common histological type of carcinomas in the larynx, and laryngeal SCC is the most frequent carcinoma among head and neck SCCs [1,2]. Accurate assessment of thyroid cartilage invasion is essential for precisely determining the extent of disease progression and tailoring appropriate treatment strategies. This evaluation plays a critical role in clinical decision making, particularly when distinguishing between organ-preserving approaches, such as radiation therapy, cordectomy or partial laryngectomy, and more radical interventions like total laryngectomy [3,4,5,6].

Conventional imaging modalities, such as computed tomography (CT) and magnetic resonance imaging (MRI), are widely used for staging laryngeal cancer. However, these techniques have limitations, particularly in accurately assessing laryngeal cartilage invasion [7,8,9]. Despite MRI generally being considered superior to CT for evaluating thyroid cartilage invasion [10,11,12], a recent study by Mohamad et al. has reported suboptimal diagnostic accuracy [13]. This study found that the overall MRI accuracy in detecting tumor invasion into the inner thyroid cortex and full-thickness thyroid cartilage was 65% and 69%, respectively. These findings raise some concerns and highlight the need to explore alternative diagnostic tools.

Among emerging imaging modalities, contrast-enhanced ultrasound (CEUS) has gained attention in oncologic imaging due to its ability to provide real-time, dynamic assessments of tissue vascularization and perfusion pattern [14,15]. Recent advancements in quantitative CEUS analysis have enabled the extraction of perfusion-based parameters, which play a crucial role in CEUS for distinguishing malignant from benign tissue and lesions [16,17,18,19,20]. Knowing that non-ossified thyroid cartilage is composed of hyaline cartilage, which is avascular, CEUS began to be explored in the scientific field, with the hypothesis that it would show no enhancement if not invaded by a tumor [21,22].

However, despite its promising potential, a limited number of studies have specifically investigated the role of qualitative CEUS in staging laryngeal cancer [23,24]. To our knowledge, no studies have investigated the role of the quantitative assessment of CEUS parameters, which offers a standardized and reproducible approach to evaluate differences in microvascular perfusion, which may be useful for the diagnostic evaluation of non-ossified thyroid cartilage invasion in laryngeal carcinoma. This study aimed to evaluate the diagnostic performance of time–intensity curve (TIC) CEUS-derived parameters in differentiating invaded from non-invaded non-ossified thyroid cartilage and comparing with histopathological assessment used as the gold standard. Our hypothesis is that these quantitative metrics can significantly enhance diagnostic efficacy and may serve as a complementary imaging modality to CT or MRI.

## 2. Materials and Methods

### 2.1. Study Design and Subjects

A prospective study was conducted at the Hospital of the Lithuanian University of Health Sciences Kauno Klinikos between 2021 and 2025. Forty-one patients with histopathologically confirmed SCC of the larynx were enrolled in this study. The inclusion criteria included the following: the availability of a contrast-enhanced computed tomography (CECT) scan demonstrating pathological infiltration adjacent to the non-ossified thyroid cartilage or clear cartilage infiltration; no history of laryngeal–hypopharyngeal surgery or chemoradiation; and surgery planned after multidisciplinary team discussion. Ten patients were excluded because some of them refused surgical treatment or did not attend further consultations or did not undergo further surgery.

All 31 male patients meeting the inclusion criteria were subjected to CEUS examination. However, 4 of them had significant motion artifacts during CEUS; therefore, data from only 27 patients were available for TIC analysis. Informed consent was obtained from all participants before the study. The study was conducted according to the guidelines of the Declaration of Helsinki. Ethical approval was obtained from Kaunas Regional Biomedical Research Ethics Committee (No. BE-2-121; dated 2021).

### 2.2. Contrast-Enhanced Ultrasound Examination

The analysis of CEUS images was performed by two radiologists with > 4 and > 20 years of experience, respectively. The radiologists were not blinded to the clinical and CECT data during the CEUS examination and image analysis.

CEUS examination was performed using a Philips Epiq 7 (expert-class) US system (Philips Healthcare, Best, The Netherlands) with a 5–12 MHz linear transducer and a mechanical index of 0.08. The target area during CEUS was selected based on CECT images, additionally using the measured distance between the lesion area of contact to the non-ossified thyroid cartilage and the upper border of the thyroid cartilage as coordinates.

During the CEUS examination, an intravenous bolus of 5 mL SonoVue (Bracco SpA, Milan, Italy), followed by saline flush, was administered. The patients were instructed to avoid swallowing or coughing during the examination. The dynamic perfusion of the suspected non-ossified thyroid cartilage invasion area and the adjacent tumor was monitored and recorded on the device hard drive for approximately 1 min. If more than one suspected invasion site was identified, the CEUS procedure was repeated after a 10 min interval.

### 2.3. Image Analysis

Recorded video loops were processed with Qlab^®^ Quantification software (Qlab 15; Philips Medical Systems, Inc., Best, the Netherlands). Both qualitative and quantitative analysis was employed for the evaluation of blood perfusion of the area of suspected non-ossified thyroid cartilage invasion and adjacent tumor.

For a qualitative analysis, the enhancement of each non-ossified thyroid cartilage area with suspected tumor invasion was evaluated with no enhancement and visible enhancement being the main categories.

**Figure 1:**
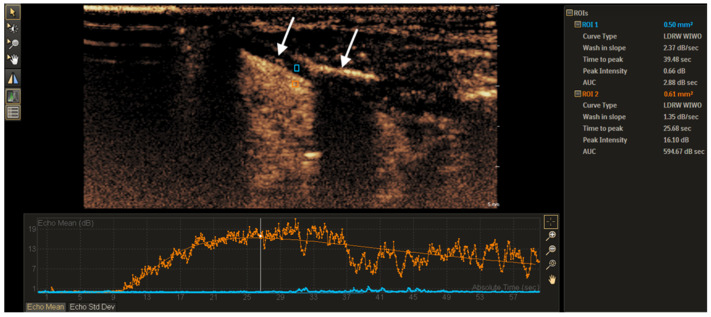
Laryngeal cancer on the left with the partially ossified thyroid cartilage (white arrows). The ROI marked by the blue rectangle frame was placed on the non-ossified thyroid cartilage and the ROI marked by the orange rectangle frame, on the adjacent tumor. Qualitative TIC analysis of wash-out was conducted by describing a wash-out curve as shallow (slow wash-out) or steep (rapid wash-out).

For quantitative analysis, the region of interest (ROI) of the tumor and the thyroid cartilage was manually determined. The first ROI was placed in the determined non-ossified thyroid cartilage area with suspected invasion, and the second comparative ROI was placed in the adjacent tumor tissue (Figure 1). The ROI size varied between 0.3 and 1.6 mm^2^ for each patient due to differences in the thickness of the non-ossified thyroid cartilage. However, for each patient, the ROI size was selected to be similar to the non-ossified cartilage and that of the region in the adjacent tumor. TICs for the tumor and the non-ossified thyroid cartilage were generated from perfusion data and analyzed with a predefined software function Auto Curve Fit control model. The following 4 parameters of quantitative analysis were produced: TTP (time to peak, s), PI (peak intensity, dB), WIS (wash-in slope, dB/s), AUC (area under curve, dB × s). Quantitative differences in TIC parameters (∆TTP, ∆PI, ∆WIS, and ∆AUC) between the tumor (t) and the suspected site of thyroid cartilage (tc) invasion were calculated, e.g., ∆PI = PI_t_ − PI_tc_. The results were written as absolute values assuming that smaller differences should show similarity with vascular tumor tissue, which could be detected in the invaded non-ossified thyroid cartilage.

### 2.4. Histological Examination

Histopathological assessment was performed by a pathologist with >20-year experience. To ensure accurate correlation between radiological findings and pathological analysis, the radiologist marked the suspected area of non-ossified thyroid cartilage invasion on an anatomical sketch of the larynx in both axial and coronal planes for each case. The macroscopic and microscopic examination was performed for each specimen. The 8th Edition of TNM Classification of Malignant Tumors [25] was used for staging.

### 2.5. Statistical Analysis

The IBM SPSS Statistics 29.0 (IBM Corp. in Armonk, NY, USA) and MedCalc 23.1.3 (MedCalc Software Ltd., Ostend, Belgium) statistical software packages were used in this study. Not all quantitative data conformed to the normal distribution. Comparisons of TIC parameters between the histopathologically proven non-ossified thyroid cartilage with and without invasion, and the tumor were performed using nonparametric tests such as the Mann–Whitney U test and the Wilcoxon test (TIC parameters of the thyroid cartilage and the tumor were treated as dependent). The non-parametric chi-square test was used to compare the enhancement of the thyroid cartilage area with and without tumor invasion. Moreover, sensitivity, specificity, and accuracy were calculated according to the formulas used in our previous work [25]. Statistically significant TIC parameters of the non-ossified cartilage with and without invasion were included in a logistic regression analysis (enter method) for calculating odds ratios (OR) with their 95% confidence intervals (CI). The area under the receiver operating characteristic (ROC) curve (AUCROC) along with sensitivity and specificity was analyzed for significant TIC parameters and their combinations. The accuracy of significant parameters was compared using the McNemar’s test. On the ROC curves, points with the greatest Youden index (sensitivity + specificity − 1) were considered the most appropriate cut-off values. A *p* value of <0.05 was considered to indicate a statistically significant difference.

## 3. Results

This prospective study involved 27 male patients with a mean age of 63.52 years (SD, 7.92; range, 49–84 years). There were 32 cases of suspected sites of non-ossified thyroid cartilage invasion analyzed by CEUS, as 5 patients had two suspected sites of invasion. There were 12 patients (44.4%) with glottic SCC and 15 patients (55.6%) with transglottic SCC with the majority showing a G2 degree of differentiation (77.8%). The patients’ distribution by pT staging was as follows: 7 (25.9%) patients had T1 SCC; 7 (25.9%) patients, T2; 9 (33.3) patients, T3; and 4 (14.8) patients, T4. In six cases (18.8%), histological proof of non-ossified thyroid cartilage invasion was obtained.

### 3.1. CEUS Imaging Features of Non-Ossified Thyroid Cartilage

**Figure 2:**
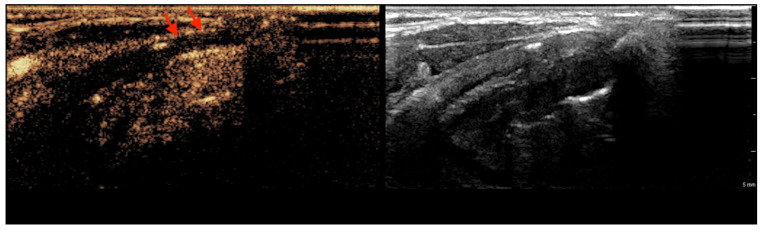
Right laryngeal SCC on CEUS. The red arrows indicate poor enhancement of thyroid cartilage. Postoperative histopathological analysis confirmed the absence of invasion.

The contrast enhancement of the non-ossified thyroid cartilage with suspected tumor invasion (according to CECT) was analyzed. The thyroid cartilage with invasion showed visible strong or moderate enhancement in 5 cases, and there was only 1 case of no enhancement. Moreover, the non-invaded cartilage did not show enhancement in 22 cases, and there were 4 cases with visible poor enhancement (*p* < 0.05) (Table 1). CEUS was able to discriminate between the invaded (visible enhancement) and the non-invaded (no enhancement) non-ossified thyroid cartilage with 83.3% sensitivity, 84.6% specificity, and 84.4% accuracy. Figure 2 is a representative CEUS scan showing poor enhancement of the thyroid cartilage.

### 3.2. TIC Analysis of Non-Ossified Thyroid Cartilage and Tumor Tissue

**Figure 3:**
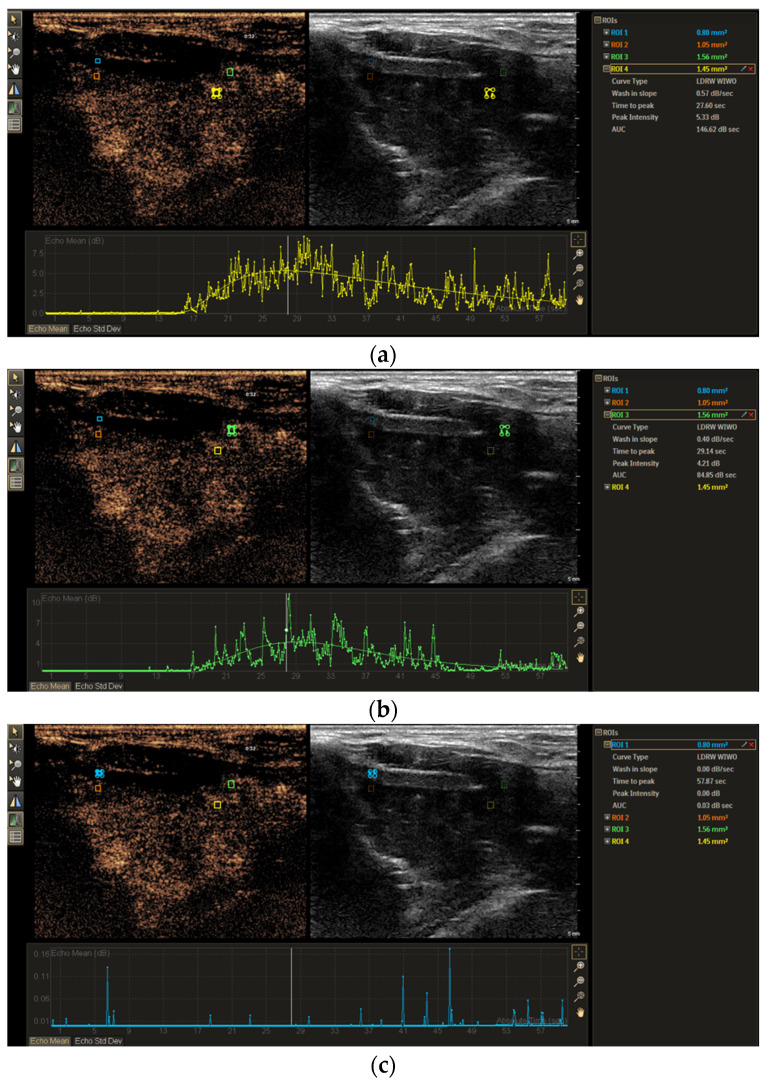
Time–intensity curve analysis of left laryngeal SCC at 28 s. (**a**) The ROI marked by the yellow rectangle frame indicates the enhancing tumor adjacent to the thyroid cartilage; (**b**) the ROI marked by the green rectangle frame shows the histologically proven invaded thyroid cartilage with enhancement; (**c**) the ROI marked by the blue rectangle frame shows non-ossified, non-enhanced cartilage without invasion.

Parameters of hemodynamics and perfusion for 3 groups of histopathologically proven tumor, invaded non-ossified thyroid cartilage, and non-invaded non-ossified thyroid cartilage were investigated and compared (Figure 3). The comparison showed statistically significant differences in TTP and PI between these 3 groups (*p* < 0.05) (Table 2). The AUC of the thyroid cartilage with and without invasion differed significantly (*p* < 0.05); however, no significant difference in the WIS was found (*p* > 0.05). AUC and WIS results differed significantly between thyroid cartilage without invasion and tumor (*p* < 0.05) and did not differ between tumor and thyroid cartilage with invasion (*p* > 0.05). Comparison of quantitative differences in TIC parameters (∆TTP, ∆PI, ∆WIS, ∆AUC) between the invaded and the non-invaded non-ossified thyroid cartilage is shown in Table 3. The median of the ∆TTP parameter in cases with thyroid cartilage invasion was statistically significantly lower than in cases with intact thyroid cartilage (*p* < 0.05).

### 3.3. TIC Morphology Analysis of Non-Ossified Thyroid Cartilage and Tumor Tissue

TICs of tumors indicated that all 32 tumor cases (100%) had shallow wash-out curves. TICs of non-ossified thyroid cartilages with and without histologically proven invasion showed that there was no statistically significant difference between wash-out curve morphology as 13 cases of thyroid cartilage without invasion and 3 cases with invasion (50% of both groups) had shallow and other 50% had steep curves (*p* > 0.05). Significant differences in the TIC wash-out shapes were observed between the tumor and non-ossified thyroid cartilage groups (*p* < 0.05).

### 3.4. Logistic Regression Analysis for Non-Ossified Thyroid Cartilage Invasion

A univariate logistic regression was carried out to determine statistically significant parameters for non-ossified thyroid cartilage invasion. Only ∆TTP was found to be statistically significant associated with non-ossified thyroid cartilage invasion. For every second increase in ∆TTP, the probability of thyroid cartilage invasion decreased by 20%. No other parameters were significant in univariate regression analysis; however, *p* values of TTP and PI were close to 0.05. Further two pairs from univariate logistic regression models of TTP + ∆TTP as Model 1 and PI + ∆TTP as Model 2 were analyzed in a multivariate logistic regression. These models did not show statistically significant results in predicting non-ossified thyroid cartilage invasion. The results of the logistic regression analysis are summarized in Table 4.

### 3.5. ROC Analysis of TIC Parameters and Their Combinations

**Figure 4:**
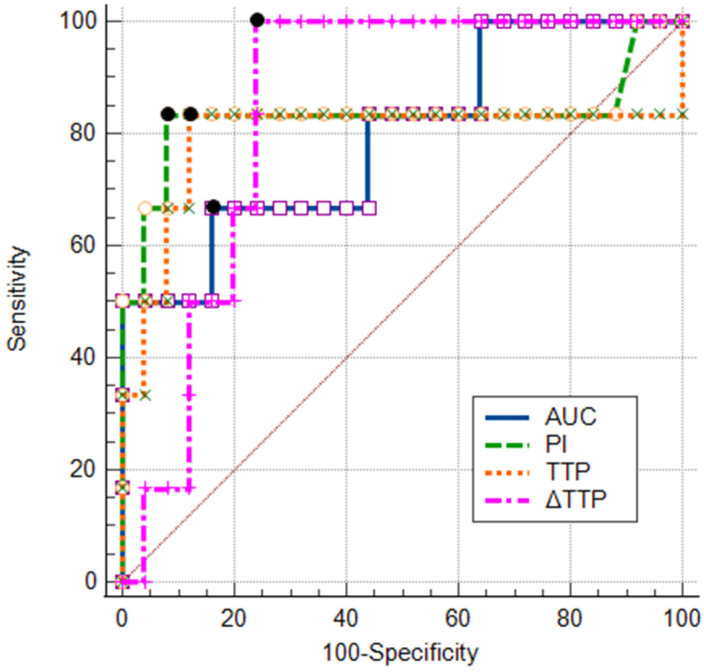
Receiver operating characteristics analysis of the time–intensity curve parameters of non-ossified thyroid cartilage with suspected tumor invasion. PI = peak intensity; AUC = area under the curve; TTP = time to peak; ∆TTP = quantitative difference in TTP between the tumor and the thyroid cartilage; black dots represent the Youden index.

Figure 4 shows the ROC curves generated for statistically significant TIC parameters (TTP, PI, AUC, and ∆TTP). Based on the greatest Youden index, the following cut-off values were obtained: ≤36.3 s for TTP, ≥3.65 dB for PI, ≥18.36 dB × s for AUC, and ≤8.9 s for ∆TTP. Diagnostic performance of all the above-mentioned parameters and their combinations (using cut-off values) were compared (Table 5). ∆TTP (AUCROC = 0.85, 95% CI: 0.68–0.95) and PI (AUCROC = 0.83, 95% CI: 0.65–0.94) were found to have the highest AUCs. ∆TTP with a higher sensitivity (100%) than specificity (76.9%) had a better ability to detect non-ossified thyroid cartilage invasion. Meanwhile, PI and TTP had better ability to exclude non-ossified thyroid cartilage invasion as their specificity reached 88.0% and 88.5%, respectively, but sensitivity was lower (83.3% each). The combination of PI and ∆TTP parameters in Model 2 increased the area under the ROC curve by 8.0% (AUCROC = 0.93, 95% CI: 0.77–0.99) when compared to the parameter with the highest AUCROC (∆TTP).

Model 2 increased the specificity (ability to exclude non-ossified thyroid cartilage invasion) to 100% and accuracy to 96.8%; however, Model 2’s accuracy did not differ significantly from that of the parameter with highest accuracy (87.5% for TTP) (*p* = 0.25).

## 4. Discussion

In recent years, CEUS has been widely studied, and its application has expanded, especially in the diagnosis and differentiation of tumor types and inflammatory diseases [14,15]. CEUS provides real-time, dynamic imaging with high spatial and temporal resolution, making it a valuable tool for assessing tumor vascularity and perfusion characteristics [26]. Its utility has been demonstrated in various oncological entities, such as liver, breast, and prostate cancers, etc. [27].

However, despite its advantages, there is no current standardized CEUS recommendation for laryngeal cancer staging according to the European Federation of Societies for Ultrasound in Medicine and Biology [14]. Given the lack of established guidelines and the limited scientific literature on this topic, we aimed to investigate the value of quantitative CEUS in assessing the invasion of the non-ossified thyroid cartilage in patients with laryngeal cancer. Specifically, our study focused on quantitative perfusion parameters such as TTP, PI, AUC, and WIS derived from a TIC analysis to explore microcirculatory changes in laryngeal SCC and the non-ossified thyroid cartilage. These findings represent a pioneering approach in utilizing CEUS to evaluate laryngeal SCC and emphasize its value in non-ossified thyroid cartilage perfusion analysis, establishing a basis for further research in this field.

In line with our expectations, we found statistically significant differences in the TIC parameters of thyroid cartilage invasion with high sensitivity and specificity, as follows: TTP (83.3%, 88.5%), PI (83.3%, 88.0%), and ∆TTP (100%, 76.9%). A visual assessment of CEUS discriminated the invaded from the non-invaded non-ossified thyroid cartilage with similar sensitivity (83.3%) and specificity (84.6%). The combination of ∆TTP and PI parameters increased the specificity, reaching 100% in excluding non-ossified thyroid cartilage invasion, while sensitivity for detecting non-ossified thyroid cartilage invasion remained the same as for PI alone. According to our results, CEUS may lead to improved pre-operative staging of the tumoral “T” component in the TNM classification of laryngeal tumors, especially in cases with equivocal CECT and/or MRI findings.

This study was based on the differences in tissue perfusion. Tumor vascularization exhibits distinct differences from normal tissue vascularization. Unlike the orderly and efficient structure of normal vasculature, tumor vessels are highly irregular, disorganized, and leaky, which results in chaotic blood flow and increased permeability. Tumor angiogenesis driven by factors such as tumor angiogenesis factor enables rapid endothelial cell division and new capillary formation, often in response to tumor-specific signals. This abnormal vascular environment can support tumor growth by facilitating nutrient delivery and waste removal, yet it also contributes to the heterogeneous microenvironment observed in a tumor [28]. Because the non-ossified cartilage is considered avascular or poorly vascularized, we can generally expect higher perfusion in areas invaded by a tumor [29,30]. This knowledge has already been employed in the evaluation of laryngeal cartilage invasion in patients with laryngeal carcinoma using multiple radiological imaging modalities [23,24,31,32,33,34,35].

Dynamic contrast-enhanced CT (DCECT), based on tissue perfusion, has been studied in the head and neck field, with the aim of achieving better diagnostic performances. DCECT uses several sequential acquisitions after intravenous contrast administration and calculated tissue perfusion parameters [35]. However, researchers found no significant added benefit of DCECT in assessing cartilage invasion, and the sensitivity of routine CECT was better than that of DCECT (85% and 75%, respectively) [35]. Some results of the latter study are in line with the findings of the study by Trojanowska et al. [33] that reported CECT being superior to DCECT (sensitivity of 66.6% and 33.3%, specificity of 96.5% of 96.5%, PPV of 80.0% and 66.6%, and NPV of 93.33% and 87.5%, respectively) in detecting malignant infiltration into the thyroid cartilage. These results suggest that DCECT is not so suitable for diagnosing laryngeal cartilage invasion as expected.

There is a growing interest in advanced MRI techniques, such as dynamic contrast-enhanced magnetic resonance imaging (DCE-MRI), a noninvasive approach used to assess microvascular perfusion by tracking changes in MRI contrast agents within targeted tissues. Since vascular irregularities are a hallmark of malignant tumors, DCE-MRI has been widely studied for its applications in cancer diagnosis, prognosis, and treatment monitoring [32,36,37,38,39,40,41]. Additionally, DCE-MRI proves to be effective in distinguishing laryngeal cartilage lesions and accurately assessing neoplastic invasion in the laryngeal cartilage [34]. According to Citil et al., the sensitivity, specificity, and accuracy of DCE-MRI for cartilage involvement was 100% [32]. However, these results need to be confirmed in larger-scale research, highlighting the necessity of further studies.

CEUS is another diagnostic tool that has been employed just in few studies for visual assessment of thyroid cartilage invasion [23,24]. They found that CEUS was superior to CECT and MRI in assessing thyroid cartilage invasion. To the best of our knowledge, we were the first to perform the quantitative analysis of laryngeal SCC and non-ossified thyroid cartilage, by extending the work of Hu et al. [23] and our previous study [24]. A study by Hu et al. reported high sensitivity, specificity, and accuracy of CEUS in the visual detection of thyroid cartilage invasion (92.9%, 87.5%, and 90.0%, respectively); meanwhile, our quantitative analysis using a combined model of ∆TTP and PI parameters showed higher specificity and accuracy (100% and 96.8%, respectively), but lower sensitivity (83.33%).

In our previous published study [24], CEUS showed a sensitivity of 100%, specificity of 84.0%, and accuracy of 87.1% in the detection of laryngeal cancer invasion into the non-ossified thyroid cartilage. In this study, due to the inclusion and exclusion of some patients, the sensitivity and accuracy of CEUS to discriminate enhancement pattern between non-invaded and invaded non-ossified thyroid cartilage decreased to 83.3% and 84.4%, respectively, while the specificity slightly increased to 84.6%. In the suspected site of cartilage invasion, visual moderate/severe accumulation of contrast agent was observed only in cases with histologically confirmed cartilage invasion. In one of the confirmed cases of cartilage invasion, no visual accumulation of contrast agent in the ROI was seen, resulting in a sensitivity of 83.3%. This could be caused by a human error in not precisely targeting the area of interest or by microinvasion of the inner cartilage plate. In 15.4% of cases without cartilage invasion, poor accumulation was observed, which could be attributed to early ossification phase, hyperemia, or mild peritumoral infiltration, leading to a specificity of 84.6%.

Based on the obtained results, we were the first to determine the values of TIC parameters for laryngeal SCC and the non-ossified thyroid cartilage. This contributes to the scientific literature for future research, as to the best of our knowledge, no similar studies have been published, thus making the comparison difficult. After performing statistical calculations, we obtained several statistically significant TIC parameters in assessing the invasion of thyroid cartilage. In our study, we identified laryngeal SCC and observed a trend demonstrating a relatively rapid accumulation (median TTP of 28.44 s). In contrast, the non-invaded non-ossified thyroid cartilage exhibited a prolonged median TTP as compared to that of the invaded thyroid cartilage (44.40 s and 32.83 s, respectively). These differences were statistically significant. Using a TTP cut-off value of 36.3 s, we can accurately differentiate (87.5%) between the invaded and the non-invaded non-ossified thyroid cartilage with a sensitivity of 83.3% and a specificity of 88.5%.

The maximum signal intensity reached with the contrast agent showed the higher mean PI value for the tumor and the invaded non-ossified thyroid cartilage than that for the non-invaded non-ossified thyroid cartilage (13.59 dB and 12.27 dB vs. 1.85 dB, respectively). The cut-off PI value for distinguishing between the non-invaded and invaded non-ossified thyroid cartilages was 3.65 dB with the sensitivity, specificity, and accuracy of 83.33%, 88.00%, and 87.1%, respectively.

We confirmed our hypothesis that the TIC parameters of the non-ossified thyroid cartilage with and without invasion differ significantly. During the study, we observed a trend indicating that the TIC parameter values of the invaded thyroid cartilage were closer to those of the tumor. Based on this observation, we decided to calculate derived values expressed as the absolute difference between the tumor and cartilage parameters. From all derivates, only ∆TTP differed significantly when comparing the thyroid cartilage without and with tumor invasion. A ROC curve analysis showed that a ∆TTP cut-off value of ≤8.9 s can accurately (81.3%) determine non-ossified thyroid cartilage invasion with a sensitivity of 100% and a specificity of 76.9%, suggesting its better ability to detect non-ossified cartilage invasion. As we hypothesized, the logistic regression analysis showed that an increase in the ∆TTP value statistically significantly decreased the probability of invasion. The combination of ∆TTP and PI slightly improved the ability to exclude non-ossified thyroid cartilage invasion with a specificity of 100%. However, an increased accuracy of this combination did not statistically significantly differ from that of the PI parameter. Our results suggest that by utilizing the main TIC parameters—∆TTP and the combination of ∆TTP, TTP, and PI—we can more confidently rule out the invasion of non-ossified thyroid cartilage.

As the first study of its kind, certain limitations are inherent. First, the study sample size was small, and it was a single-center study. Second, the technique is highly operator-dependent, requiring an experienced professional in CEUS as well as in MRI and CT imaging. Third, motion artifacts were a challenge in patients with an advanced disease as they struggled to maintain shallow breathing or to remain still in a supine position for extended periods. Additionally, the accurate identification of ROI (according to CECT) posed difficulties, and perfusion data were derived from a single tissue slice, which may limit generalizability. Finally, anatomical variations, such as a thin, non-ossified thyroid cartilage, could have influenced TIC parameters due to the small ROI size, increasing sensitivity to movement.

In summary, this study is the first to address the CEUS quantitative analysis of the evaluation of non-ossified thyroid cartilage invasion in patients with laryngeal cancer, providing a critical starting point for further research. Understanding that further research on CEUS is needed, it should focus on larger patient samples and should ideally adopt a multicentric approach to enhance reliability. Additionally, exploring the feasibility of fusing CEUS images with MRI or CT scans may further improve diagnostic accuracy and clinical utility.

## 5. Conclusions

The results obtained enhance the diagnostic value of the CEUS examination by assessing and utilizing quantitative parameters, particularly a combination of ∆TTP and PI, to rule out and detect non-ossified thyroid cartilage invasion. Our study highlights CEUS as a suitable and easily accessible complementary method to MRI or CT for cases where non-ossified thyroid cartilage invasion remains unclear.

## Figures and Tables

**Table 1:** Contrast enhancement of the non-ossified thyroid cartilage with suspected tumor invasion.

Feature	Thyroid Cartilage Without Invasion (*n* = 26)	Thyroid Cartilage with Invasion (*n* = 6)	*p* Value
No enhancement	22 (84.6)	1 (16.7)	<0.05
Visible enhancement	4 (15.4)	5 (83.3)	

Values are number (percentage).

**Table 2:** TIC parameters of the tumor and the thyroid cartilage with and without invasion.

Parameter	Thyroid Cartilage Without Invasion	Thyroid Cartilage with Invasion	*p* Value ^1^	Tumor	*p* Value ^2^
TTP, s	44.4 (30.99–58.31)	32.83 (29.02–58.70)	<0.05	28.44 (10.95–49.80)	<0.05 ^n^ <0.05 ^i^
PI, dB	1.85 (0.00–7.22)	12.27 (0.30–21.80)	<0.05	13.59 (4.65–46.41)	<0.05 ^n^ <0.05 ^i^
AUC, dB × s	10.30 (0.30–58.54)	69.05 (3.85–330.12)	<0.05	313.00 (61.05–657.89)	<0.05 ^n^ >0.05 ^i^
WIS, dB/s	3.64 (0.00–19.51)	11.89 (0.60–46.77)	>0.05	1.74 (0.48–10.06)	<0.05 ^n^ >0.05 ^i^

Values are median (min–max). Abbreviations: TTP = time to peak; PI = peak intensity; AUC = area under curve; WIS = wash-in slope. ^1^ Comparisons made using Mann–Whitney *U* test. ^2^ Comparisons made using Wilcoxon test. ^n^ Thyroid cartilage without invasion; ^i^ thyroid cartilage with invasion.

**Table 3:** Quantitative differences in TIC parameters (∆TTP, ∆PI, ∆WIS, and ∆AUC) between the tumor and the non-invaded or the invaded non-ossified thyroid cartilage.

Parameter	Thyroid Cartilage Without Invasion	Thyroid Cartilage with Invasion	*p* Value
∆TTP, s	15.19 (2.02–36.54)	5.10 (1.83–8.90)	<0.05
∆PI, dB	11.60 (3.33–37.11)	4.15 (1.60–24.61)	>0.05
∆AUC, dB × s	333.75 (67.18–650.42)	199.55 (85.39–303.46)	>0.05
∆WIS, dB/s	5.11 (0.24–15.92)	11.18 (0.27–45.87)	>0.05

Values are absolute and expressed as median (min–max). Abbreviations: TTP = time to peak; PI = peak intensity; AUC = area under the curve; WIS = wash-in slope.

**Table 4:** Logistic regression for diagnosis of non-ossified thyroid cartilage invasion.

Univariate Logistic Regression		Odds Ratio	95% CI	*p* Value
TTP (1 s)		0.88	0.76–1.01	0.067
PI (1 dB)		1.59	0.97–2.62	0.067
AUC (1 dB × s)		1.03	0.99–1.07	0.103
∆TTP (1 s)		0.80	0.64–1.00	0.047
Multivariate logistic regression models				
Model 1	TTP (1 s)	1.04	0.87–1.25	0.64
	∆TTP (1 s)	0.76	0.56–1.05	0.09
Model 2	PI (1 dB)	1.66	0.87–3.14	0.12
	∆TTP (1 s)	0.82	0.64–1.06	0.12

Abbreviations: TTP = time to peak; PI = peak intensity; AUC = area under curve.

**Table 5:** Diagnostic performance of TIC perfusion parameters in predicting thyroid cartilage invasion.

Parameter	AUCROC	95% CI	Sensitivity (%)	Specificity (%)	Accuracy (%)	Cut-Off	*p* Value
TTP (s)	0.80	0.62–0.92	83.3	88.5	87.5	≤36.3	0.047
PI (dB)	0.83	0.65–0.94	83.3	88.0	87.1	≥3.65	0.015
AUC (dB × s)	0.79	0.61–0.92	66.7	80.0	77.4	≥18.36	0.008
∆TTP (s)	0.85	0.68–0.95	100.0	76.9	81.3	≤8.9	<0.001
Model 1 (TTP + ∆TTP)	0.82	0.65–0.93	88.5	76.9	87.5	–	<0.0001
Model 2 (PI + ∆TTP)	0.93	0.77–0.99	83.3	100	96.8	–	<0.0001

Abbreviations: TTP = time to peak; PI = peak intensity; AUC = area under curve; AUCROC = area under the receiver operating characteristic curve.

## Data Availability

The data that support the findings of this study are available from the corresponding author upon reasonable request. The data are not publicly available due to privacy or ethical restrictions.

## Abbreviations

The following abbreviations are used in this manuscript:

SCC	Squamous cell carcinoma
CT	Computed tomography
MRI	Magnetic resonance imaging
CEUS	Contrast-enhanced ultrasound
TIC	Time–intensity curve
CECT	Contrast-enhanced computed tomography
ROI	Region of interest
TTP	Time to peak
PI	Peak intensity
WIS	Wash-in slope
AUC	Area under the curve
DCECT	Dynamic contrast-enhanced computed tomography
ROC	Receiver operating characteristic
